# Trends in Health Care Use Among Black and White Persons in the US, 1963-2019

**DOI:** 10.1001/jamanetworkopen.2022.17383

**Published:** 2022-06-14

**Authors:** Samuel L. Dickman, Adam Gaffney, Alecia McGregor, David U. Himmelstein, Danny McCormick, David H. Bor, Steffie Woolhandler

**Affiliations:** 1 Texas Policy Evaluation Project, The University of Texas at Austin, Austin; 2 Planned Parenthood South Texas, San Antonio, Texas; 3 Division of Pulmonary and Critical Care Medicine, Harvard Medical School/Cambridge Health Alliance, Cambridge, Massachusetts; 4 Department of Health Policy and Management, Harvard T.H. Chan School of Public Health, Boston, Massachusetts; 5 City University of New York at Hunter College, New York, New York; 6 Department of Medicine, Harvard Medical School/Cambridge Health Alliance, Cambridge, Massachusetts; 7 Public Citizen Health Research Group, Washington, DC

## Abstract

**Importance:**

In the US, Black people receive less health care than White people. Data on long-term trends in these disparities, which provide historical context for interpreting contemporary inequalities, are lacking.

**Objective:**

To assess trends in Black-White disparities in health care use since 1963.

**Design, Setting, and Participants:**

This cross-sectional study analyzed 29 US surveys conducted between 1963 and 2019 of noninstitutionalized Black and non-Hispanic White civilians.

**Exposures:**

Self-reported race and ethnicity.

**Main Outcomes and Measures:**

Annual per capita visit rates (for ambulatory, dental, and emergency department care), inpatient hospitalization rates, and total per capita medical expenditures.

**Results:**

Data from 154 859 Black and 446 944 White (non-Hispanic) individuals surveyed from 1963 to 2019 were analyzed (316 503 [52.6%] female; mean [SD] age, 37.0 [23.3] years). Disparities narrowed in the 1970s in the wake of landmark civil rights legislation and the implementation of Medicare and Medicaid but subsequently widened. For instance, the White-Black gap in ambulatory care visits decreased from 1.2 (95% CI, 1.0-1.4) visits per year in 1963 to 0.8 (95% CI, 0.6-1.0) visits per year in the 1970s and then increased, reaching 3.2 (95% CI, 3.0-3.4) visits per year in 2014 to 2019. Even among privately insured adults aged 18 to 64 years, White individuals used far more ambulatory care (2.6 [95% CI, 2.4-2.8] more visits per year) than Black individuals in 2014 to 2019. Similarly, White peoples’ overall health care use, measured in dollars per capita, exceeded that of Black people in every year. After narrowing from 1.96 in the 1960s to 1.26 in the 1970s, the White-Black expenditure ratio began widening in the 1980s, reaching 1.46 in the 1990s; it remained between 1.31 and 1.39 in subsequent periods.

**Conclusions and Relevance:**

This study’s findings indicate that racial inequities in care have persisted for 6 decades and widened in recent years, suggesting the persistence and even fortification of structural racism in health care access. Reform efforts should include training more Black health care professionals, investments in Black-serving health facilities, and implementing universal health coverage that eliminates cost barriers.

## Key Points

**Question:**

How much progress has been made during the past 6 decades in equalizing access to health care for Black and White people in the US?

**Findings:**

In this repeat cross-sectional analysis using 6 decades of national data, Black-White inequalities in health care use, measured as visit rates or total health care expenditures, narrowed after the implementation of Medicare and Medicaid but subsequently widened.

**Meaning:**

These findings suggest that policy changes are needed to address entrenched racial inequalities in the US health care system.

## Teaser

This cross-sectional study examines disparities in health care use between Black and White people as measured by health care expenditures and visit rates between 1963 and 2019.

## Introduction

Before the passage of landmark civil rights legislation and the implementation of Medicare and Medicaid in the mid-1960s, access to medical care was sharply restricted, particularly for Black and poor people.^1^ Subsequently, de jure segregation in health facilities was outlawed, and gaps in insurance coverage narrowed.^2,3^ However, lack of health insurance has remained more common among Black people than White people, even after implementation of the Patient Protection and Affordable Care Act (ACA).^4^ Moreover, Black people are less likely to have private health coverage, which often facilitates preferential access to medical care.^5^

Black people in the US have higher rates of chronic conditions, such as diabetes^6^ and hypertension,^7^ and shorter life expectancy than their White counterparts.^8^ Hence, ambulatory medical care use, if allocated solely based on need, would be higher for Black people. Several studies have documented Black-White inequalities in the use of ambulatory and dental care^9^ and for specific health services^10,11^ and age groups.^12^ However, little information is available on more recent disparities in health care use or on long-term trends in disparities spanning the period since Medicaid and Medicare were introduced. Using data from nationally representative surveys, we assessed 6 decades of racial disparities in medical care use, as measured by health care expenditures and visit rates.

## Methods

### Data Sources

We aligned and analyzed data from 29 nationally representative surveys of health care use and expenditures by and on behalf of non-Hispanic Black and non-Hispanic White individuals of all ages in the civilian, noninstitutionalized US population. (The surveys’ definitions of Hispanics and other racial and ethnic groups changed over time, precluding accurate assessment of time trends for those groups, which we excluded from analyses.) The Cambridge Health Alliance’s institutional review board does not consider analyses of publicly available data human subjects research, so patient informed consent was waived. This study followed the Strengthening the Reporting of Observational Studies in Epidemiology (STROBE) reporting guideline.

The surveys, conducted starting in 1963, included the 1963 and 1970 Surveys of Health Services Utilization and Expenditures (N = 7759 and 11 619, respectively); 1977 National Medical Care Expenditure Survey (N = 38 815); 1980 National Medical Care Utilization and Expenditure Survey (N = 17 123); 1987 National Medical Expenditure Survey (N = 34 459); and the continuous 1996-2019 Medical Expenditure Panel Surveys, conducted annually (N = 21 571-37 418). Each survey collected information on participants’ demographic characteristics, self-reported health status, use of health care services, and health care expenditures (including all out-of-pocket and third-party insurance payments). Respondents’ self-reported health care expenditures were verified with hospitals, physicians, and other medical facilities in all surveys except the 1980 National Medical Expenditure Survey, which instead verified spending reports with corresponding Medicare and Medicaid claims and eligibility data.

We assessed 2 types of health care use metrics: counts of visits and inpatient days and total (ambulatory and inpatient) use measured in dollars (ie, total expenditures for care). Data on the number of ambulatory medical visits were collected in all survey years. Counts of inpatient days and dental visits were first reported in 1970, and emergency department visits were first reported in 1977.

Data on overall health care use measured by expenditures for care were available in all surveys. The surveys between 1963 and 1987 recorded costs based on charges (including the value of all uncompensated care) rather than actual payments; at the time, charges more closely corresponded to actual payments received than they currently do.^13,14^ Subsequent surveys reported expenditures based on payments. Since 1996, the Medical Expenditure Panel Surveys has included the dollar value of uncompensated care at public clinics and hospitals in tabulations of expenditures, while assigning zero expenditures to uncompensated care delivered by private hospitals or other private medical facilities. Survey questions remained substantially consistent across surveys, permitting time-trend analyses of health care use and expenditure, as in previous studies.^15,16^

### Data Analysis

Data analyses were conducted between July 2, 2022, and December 4, 2022. We delineated trends in 4 count-based measures of use: visits to ambulatory medical practitioners, visits to dentists, visits to emergency departments, and hospital use (measured as inpatient days). In addition, we tabulated total health expenditures, including all out-of-pocket and third-party payments (eg, insurance payments) by and on behalf of Black and White survey respondents. We excluded Hispanic participants (of either race) in all surveys except the 1963 survey, which did not record Hispanic ethnicity.

Our main analyses tabulated unadjusted per capita means, with dollar figures adjusted to 2019 dollars using the Gross Domestic Product Price Index.^17,18^ We also report the White-Black ratio for each health care use measure and differences for each outcome stratified by age group: children, adults aged 18 to 64 years, and adults 65 years or older. To explore whether White-Black disparities were attributable solely to insurance coverage, we repeated all analyses in samples restricted to privately insured adults aged 18 to 64 years and adults aged 18 to 64 years with Medicaid. In addition, to determine whether age and sex differences explain present-day disparities in health care use, we conducted multivariable regressions using pooled 2014 to 2019 data (to improve precision) and estimated White-Black differences among all adults aged 18 to 64 years, privately insured adults aged 18 to 64 years, and Medicaid-insured adults aged 18 to 64 years. All of these analyses were adjusted for age (in years) and sex (male or female).

Because underlying health differences could confound race-related differences in health care use, we conducted sensitivity analyses adjusting for self-reported health status (poor, fair, good, very good, or excellent). We also explored whether differences in use might be mediated by race-related differences in socioeconomic status (SES) by conducting analyses that included 2 SES indicators: health care coverage and family income as a percentage of the federal poverty level. We calculated the percentage of the overall association of race that was mediated by each of these SES variables (calculating the associations of each SES measure separately and combined), while controlling for underlying age and sex differences that could confound the association of SES on health care use.

### Statistical Analysis

We analyzed count outcomes using negative binomial regression and expenditures using linear regression.^19^ Differences were considered significant at *P* < .05 using 2-tailed tests. Because health expenditures are highly skewed, we also performed sensitivity analyses using log-transformed expenditures. To assess whether a small number of very high-cost patients drove our results, we repeated multivariable analyses using quantile regression at the 50th, 75th, 90th, 95th, and 97.5th percentiles.^20^ We performed separate analyses for each data year. However, to simplify presentation of our findings, we present the mean for each decade from the 1960s through 2000s, and for the pre- (2010-2013) and post- (2014-2019) ACA periods. All estimates incorporate person-level weights that allow extrapolation to the US noninstitutionalized population. The 1963 Survey of Health Services Utilization and Expenditures used a simple random (equal-weight) sample. For all other surveys, we used SAS software, version 9.4 (SAS Institute Inc) and Stata software, version 16.1 (StataCorp LLC) procedures that account for complex survey designs.

## Results

We analyzed data from 154 859 Black and 446 944 White (non-Hispanic) individuals surveyed from 1963 to 2019 (316 503 [52.6%] female; mean [SD] age, 37.0 [23.3] years). White peoples’ overall health care use (measured as expenditures by or on behalf of individuals) exceeded that of Black people in every year (Figure). After initially narrowing from 1.96 in the 1960s to 1.26 in the 1970s, the White-Black expenditure ratio began widening in the 1980s, reaching 1.46 in the 1990s; it remained between 1.31 and 1.39 in subsequent periods (eTable 1 in the Supplement). The absolute White-Black per capita expenditure gap was $1880 in 2014 to 2019, larger than during any other period.

**Figure.**
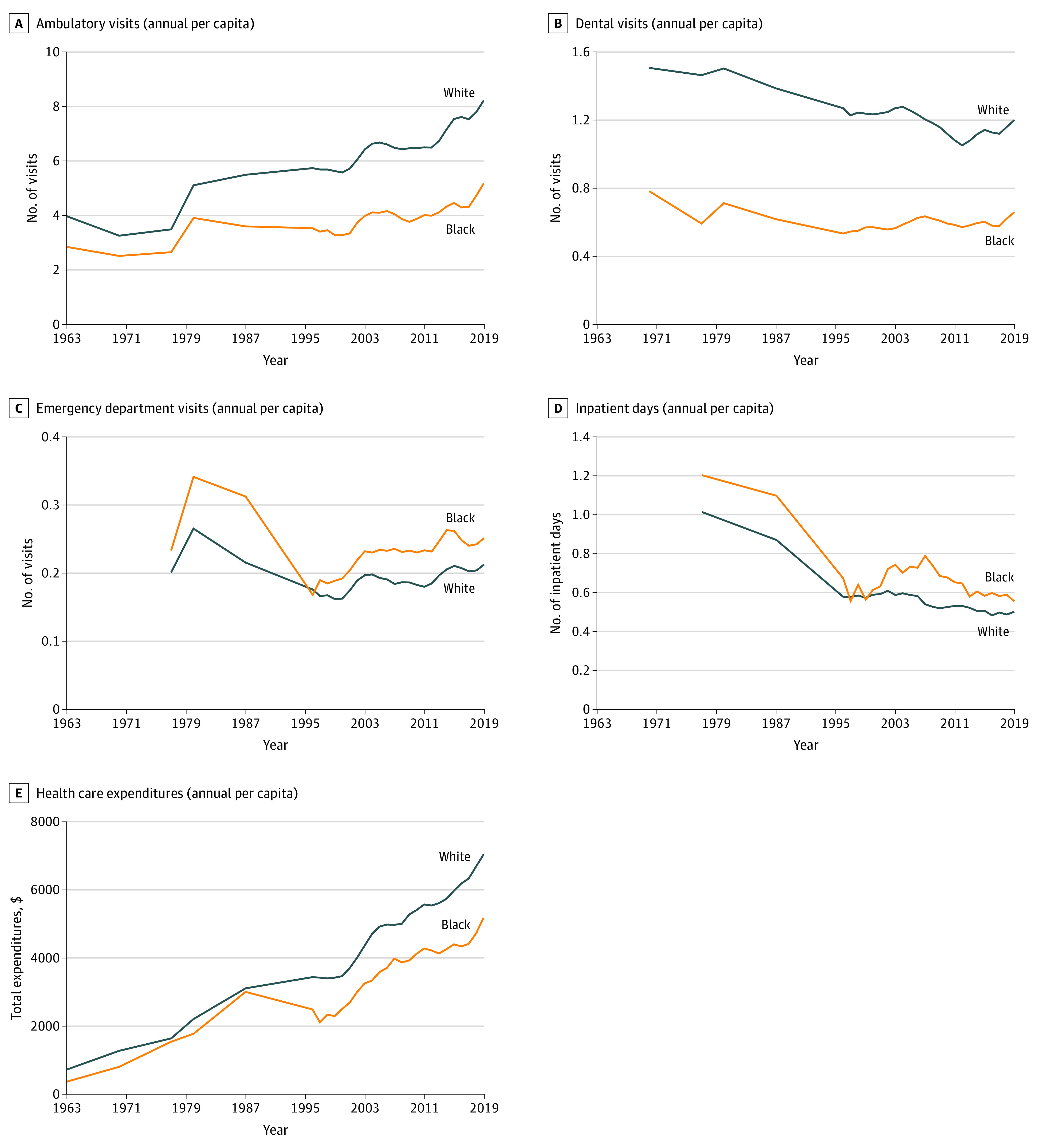
Black-White Disparities in Annual per Capita Visits, Inpatient Days, and Expenditures, 1963-2019 Data sources: 1963 and 1970 Surveys of Health Services Utilization and Expenditures, 1977 and 1980 National Medical Care Utilization and Expenditure Surveys, 1987 National Medical Expenditure Survey, and 1996-2019 Medical Expenditure Panel Surveys. Estimates are adjusted for complex survey design using SAS 9.4 survey procedures, and lines are smoothed using 3-year moving average for annual surveys (1996-2019).

Rates of ambulatory care visits followed the same temporal pattern; the White-Black gap in use decreased from 1.2 (95% CI, 1.0-1.4) in 1963 to 0.8 (95% CI, 0.6-1.0) in the 1970s and began increasing in the 1980s, reaching 3.2 (95% CI, 3.0-3.4) visits per year in 2014 to 2019. In contrast, large disparities in dental visits increased between the 1970s and 1990s (when White people had 113% and 123% more dental visits per capita than Black people, respectively), then narrowed slightly to 86% (95% CI, 80%-91%) in 2014 to 2019. As in the past, Black people currently have slightly more emergency department visits per year (0.04; 95% CI, 0.03-0.05) than White people and spend slightly more days (0.09; 95% CI, 0.02-0.15) as inpatients in hospitals. eTable 1 in the Supplement gives the mean per capita use per year for the Black and White populations for each decade of the study.

Restricting analyses to privately insured adults 18 to 64 years of age modestly attenuated White-Black disparities: White individuals used 2.6 (95% CI, 2.4-2.8) more ambulatory visits per year than Black individuals in 2014 to 2019 (eTable 1 in the Supplement). Disparities in use were more pronounced among women, particularly for ambulatory and dental visits (eTable 2 in the Supplement).

In the post-ACA period (2014-2019), large racial disparities in ambulatory care visits, dental visits, and total expenditures persisted before (eTable 1 in the Supplement) and after age and sex adjustment (Table 1). In adjusted analyses, Black people had 2.63 (95% CI, 2.31-2.95) fewer ambulatory care visits per year, 0.52 (95% CI, 0.48-0.56) fewer dental visits, and $992 (95% CI, $678-$1306) lower total health expenditures than White people. In SES mediation analyses (controlling for age and sex), the indirect association of income and health coverage (combined) was large for Black-White gaps in emergency department visits (accounting for 88.3% of the direct race association) and inpatient days (93.5%), whereas gaps for ambulatory care visits, dental visits, and total health expenditures were only modestly mediated (by 0.1% for ambulatory care visits, 23.5% for dental visits, and −26.1% for total health expenditures) (Table 2).

**Table 1.** Association Between Annual per Capita Health Care Use and Race, Adjusted for Age and Sex, 2014-2019^a^

Health care use	Adjusted per capita difference, Black – White (95% CI)^b^			
	Total population	Adults aged 18-64 y	Adults aged 18-64 y	
			Privately insured	Medicaid
Ambulatory care visits, No.	−2.63 (−2.95 to −2.31)^c^	−2.38 (−2.71 to −2.04)^c^	−2.46 (−2.82 to −2.10)^c^	−4.87 (−6.46 to −3.28)^c^
Dental visits, No.	−0.52 (−0.56 to −0.48)^c^	−0.42 (−0.47 to −0.38)^c^	−0.38 (−0.44 to −0.33)^c^	−0.32 (−0.40 to −0.24)^c^
Emergency department visits, No.	0.05 (0.04 to 0.07)^c^	0.07 (0.06 to 0.09)^c^	0.04 (0.02 to 0.05)^c^	−0.02 (−0.08 to 0.03)
Inpatient days, No.	0.13 (0.04 to 0.23)^c^	0.15 (0.07 to 0.23)^c^	0.09 (0.00 to 0.18)^c^	−0.14 (−0.64 to 0.36)
Total health expenditures, $	−992 (−1306 to −678)^c^	−889 (−1281 to −497)^c^	−1154 (−1675 to −632)^c^	−1453 (−2654 to −252)^c^

^a^ Data are pooled from the 2014 to 2019 Medical Expenditures Panel Surveys. The 95% CIs are adjusted for complex survey design using SAS software, version 9.4 survey procedures. Negative binomial regressions were used for count outcomes (eg, visits) and linear regression for expenditures.

^b^ Differences are adjusted for age (in years) and sex (male and female) using SAS software, version 9.4 survey procedures.

^c^ Difference is significant at *P* < .05.

**Table 2.** Mediation Analysis: Association of SES Variables With Black-White Differences in Health Care, 2014-2019

Health care use	Direct association of race (Black − White, point estimate with no SES mediators)^a^	Point estimate of race association in regression model with specified SES mediator^a^			Direct race association mediated by specified SES mediator, %			
		Income	Insurance	Income and insurance	Income^b^	Insurance^c^	Income and insurance
Ambulatory care visits, No.	−2.630	−2.505	−2.740	−2.628	4.8	−4.2	0.1
Dental visits, No.	−0.521	−0.443	−0.450	−0.398	15.0	13.7	23.5
Emergency department visits, No.	0.053	0.020	0.023	0.006	63.2	57.7	88.3
Inpatient days, No.	0.133	0.040	0.057	0.009	70.1	57.1	93.5
Total health expenditures, $	−992	−1130	−1177	−1251	−13.9	−18.7	−26.1

^a^ Point estimates indicate annual per capita rates (Black − White) adjusted for age and sex.

^b^ Income was specified as a continuous percentage of the federal poverty level, which accounts for family size and composition.

^c^ Insurance was categorized as in the surveys (uninsured, insured with public coverage, or insured with private coverage).

Analyses limited to adults aged 18 to 64 years with private insurance or Medicaid showed similar disparities, with White-Black expenditure gaps of $889 per capita among those with private insurance and $1154 per capital among those with Medicaid. Multivariable models with additional adjustment for health status (eTable 3 in the Supplement) yielded similar results, although disparities in health care use were slightly larger than in models adjusted only for age and sex.

In age-stratified analyses (Table 3), White-Black disparities in ambulatory care visit rates increased in all age groups between the 1980s and the post-ACA period, when White children had 1.99 more ambulatory care visits per capita, adults aged 18 to 64 years had 2.30 more visits, and older adults had 4.17 more visits than similar-aged Black people. Although absolute differences in visits were highest among older adults (reflecting their higher use overall), that group had a smaller relative White-Black gap (42% more visits for White respondents) than among children (a 75% difference) or adults aged 18 to 64 years (a 49% difference). Racial gaps in total health expenditures were smaller among older adults ($806 absolute difference) than for adults aged 18 to 64 years ($1224) or children ($1205).

**Table 3.** Differences Between White and Black Individuals in the US in per Capita Ambulatory Care Visits and Total Health Expenditures by Age Group, 1960s-2014/2019^a^

Age group, y	Differences in health care use per capita (Black − White)						
	1960s	1970s	1980s	1990s	2000s	2010s	
						2010-2013	2014-2019
**Ambulatory care visits, No. (95% CI)**							
0-17	−1.32 (−1.68 to −0.98)^b^	−1.12 (−1.22 to −1.02)^h^	−2.16 (−2.43 to −1.88)^b^	−1.79 (−2.00 to −1.59)^b^	−1.78 (−1.92 to −1.64)^b^	−1.73 (−2.04 to −1.41)^b^	−1.99 (−2.46 to −1.52)^b^
18-64	−0.88 (−1.63 to −0.14)^b^	−0.44 (−0.60 to −0.28)^b^	−0.93 (−1.41 to −0.46)^b^	−1.87 (−2.20 to −1.54)^b^	−1.83 (−2.05 to −1.60)^b^	−1.74 (−2.08 to −1.39)^b^	−2.30 (−2.62 to −1.99)^b^
≥65	−1.59 (−0.88 to 4.06)^b^	0.70 (0.39 to 1.00)^b^	−0.13 (−1.77 to 1.51)	−2.15 (−3.09 to −1.22)^b^	−3.07 (−3.82 to −2.31)^b^	−3.68 (−4.61 to −2.75)^b^	−4.17 (−5.02 to −3.32)^b^
**Total health expenditures (95% CI), $**							
0-17	−242 (−290 to −194)^b^	−338 (−385 to −291)^b^	−531 (−789 to −273)^b^	−917 (−1353 to −481)^b^	−1061 (−1299 to −823)^b^	−804 (−1386 to −221)^b^	−1205 (−1695 to −715)^b^
18-64	−350 (−544 to −157)^b^	−39 (−197 to 119)	424 (69 to 778)^b^	−718 (−964 to −472)^b^	−589 (−815 to −362)^b^	−757 (−1132 to −382)^b^	−1224 (−1635 to −812)^b^
≥65	−371 (−769 to 27)	−158 (−643 to 327)	433 (−1154 to 2021)	−18 (−997 to 1032)	−47 (−803 to 709)	−643 (−1613 to 326)	−806 (−1848 to 236)

^a^ Data are from the 1963 and 1970 Surveys of Health Services Utilization and Expenditures, 1977 and 1980 National Medical Care Utilization and Expenditure Surveys, 1987 National Medical Expenditure Survey, and 1996-2019 Medical Expenditure Panel Surveys. Data represent difference in mean visit numbers and mean total expenditures (Black − White).

^b^ Difference is significant at *P* < .05.

Quantile regression results appear in eTable 4 in the Supplement. These analyses indicate that White-Black differences were most extreme among the highest users. White patients had 8.33 (95% CI, 7.87-8.79) more ambulatory care visits at the 95th percentile of ambulatory care use and 11.34 (95% CI, 10.18-12.50) more visits at the 97.5th percentile of ambulatory care use than Black patients. Somewhat smaller but still significant differences were present among persons at the median of health care use. These, Black-White gaps remained statistically significant in regression models using log-transformed expenditures (eTable 5 in the Supplement).

## Discussion

This repeat cross-sectional study found that large racial differences in the use of medical care that were present in the early 1960s narrowed in the following decade but have widened since then. The White-Black gap in expenditures is at an all-time high, as measured by inflation-adjusted dollars, and both absolute and relative disparities in ambulatory care visit rates are larger today than in 1963. Now, as in the past, Black people in the US experience a greater burden of ill health, suggesting that care is distributed inversely to need.

Although observational studies cannot prove causation, the attenuation of disparities in health care use after 1963 coincided with the implementation of Medicare and Medicaid (which outlawed segregation in medical facilities), the advent of community health centers, and new civil rights protections that improved Black individuals’ access to housing, jobs, education, and the ballot box. The divergence starting in the 1980s coincided with waning civil rights enforcement, increasing incarceration (especially for Black men), and stagnating Medicaid enrollment and expenditures (as a share of national health expenditures).^21,22^ Although the ACA’s 2014 implementation narrowed racial disparities in insurance coverage and self-reported access to care,^23,24^ we found that gaps in health care use persisted. Previous analyses of health care use^9,25^ found similar gaps in recent years; our analysis adds several decades of historical data to these findings, contextualizing these disparities within earlier changes in health care financing, such as the rollout of Medicare and Medicaid beginning in the mid-1960s and private insurers’ increases in patient cost-sharing since the 1990s. The current White-Black gap in ambulatory care visit rates is larger than ever before, and differences in expenditures are wider than in the pre-ACA period and at an all-time high measured by real (inflation-adjusted) dollars.

The racial differences we found in health care use could represent underuse by Black individuals, overuse by White individuals, or both. Overtreatment (often referred to as low-value care) cost $210 billion (approximately 10.0% of personal health spending) in 2009 according to one estimate^26^ or, according to others, as much as to 2.6% of overall expenditures^27^ and 2.7% of Medicare payments.^28^ If the highest estimate is correct, overuse could explain approximately half of the racial difference in expenditures, but only if all overuse occurred among White patients. However, studies suggest that disadvantaged patients also receive low-value services. For instance, Medicaid patients receive low-value care at similar rates to privately insured persons,^29^ and Black and Hispanic Medicare beneficiaries receive some low-value services at higher rates than White beneficiaries.^30^

Previous studies suggest that at least part of the racial gap in health care use is attributable to underuse of needed care by Black individuals. Compared with non-Hispanic White individuals, Black individuals are more frequently hospitalized for ambulatory care sensitive conditions (a marker for inadequate outpatient care),^31,32^ and Black persons with hypertension (a major contributor to all-cause mortality) are less likely to have their blood pressure controlled.^7^ Relative underuse by Black people has also been found for knee replacements,^33^ optimal surgery for lung cancer^34^ and laryngeal cancer,^35^ mental health and substance use treatment,^11^ neurologist care for complex conditions,^10^ and prenatal and postpartum care.^36^ The slightly greater use of emergency department visits and inpatient hospital days among Black individuals may be attributable, in part, to their deficit of ambulatory care.

Financial barriers may disproportionately reduce Black peoples’ use of care. A much greater percentage of Black than White people in the US are uninsured,^37^ and more Black adults report skipping needed care because of costs.^38^ White-Black gaps in visit rates narrowed in the first year after Medicare was implemented,^39^ and enrollment in the State Children’s Health Insurance Program reduced racial differences in use and access to ambulatory care.^40^ Our finding that racial disparities in outpatient visits and total expenditures were attenuated among older adults (most of whom are covered by Medicare) and among working-age adults with private coverage also suggests the salience of lowering financial barriers and the potential importance of payment reform (ie, equalizing fees that hospitals, physicians, and other medical facilities receive for patients of different racial and ethnic groups) as strategies to reduce disparities.

However, the fact that racial disparities remained when we limited our analyses to persons with private insurance, or to Medicaid enrollees or older adults, points to factors other than insurance coverage. Copayments and deductibles may be a higher hurdle for privately insured Black people in the US, whose mean family incomes are 27% lower than the incomes of privately insured Whites (D.U.H., unpublished analysis of the March 2019 Current Population Survey, April 10, 2020, and May 8, 2022). For those with Medicaid, which imposes few out-of-pocket costs, non–payment-related factors associated with structural racism may obstruct access to care, a possibility supported by our finding that inclusion of income as a mediator in our models failed to attenuate Black-White gaps in ambulatory care visits in recent years. Unmeasured factors, such as household wealth, residential and occupational segregation (which likely increases distance from practitioners), inadequate transportation, or inability to take time off from work, may contribute to racial disparities. Psychosocial factors, such as differences in cultural norms and Black patients’ beliefs that they may be unwelcome, will face bias, or cannot trust the health care system may reduce their care-seeking and health care use.^41^ Some medical practitioners’ implicit racial bias may cause them to make fewer needed referrals to specialists.^42^ Finally, the paucity of Black medical practitioners may also discourage Black patients from complying with recommendations for follow-up visits, testing, and other care.^43^

### Limitations

Several caveats apply to our findings. Although the surveys we analyzed were designed to accurately reflect national trends, they all understate total visits and expenditures because patients with high expenditures and high health care use are underrepresented, and people in nursing homes, other institutions (eg, jails and prisons), and the military are excluded. In addition, visits to alternative medicine practitioners (eg, acupuncturists) are excluded from ambulatory care visit counts in some of the surveys.^16,44,45^ The 1963, 1970, 1977, and 1980 surveys recorded charges instead of actual payments for medical services, which were slightly higher than actual payments, although overestimation of costs was likely similar for White and Black respondents. Our dollar estimates after 1980 (although not our counts of visits or inpatient days) exclude free care delivered outside the public sector; a previous analysis^46^ suggests a negligible risk of bias from this exclusion. Although highly skewed data, such as health expenditures, theoretically limit the validity of linear regression analyses, such analyses using data sets with large sample sizes are generally reliable and easier to interpret than results from alternative approaches.^19^

Disparities in aggregate expenditures are influenced by payment rates, which are usually highest for private insurance. Hence, the expenditure disparities we found are likely in part due to higher rates of Medicaid coverage and uninsurance (and lower payment per care episode) for Black individuals.^47^ However, the large disparities in visit rates indicate that differences in prices cannot fully account for the disparities in expenditures.

The 1963 survey (when Hispanic people accounted for <3% of the White population)^48^ instructed interviewers to classify persons of Spanish or Mexican descent as White and did not separately record Hispanic ethnicity. Hence, we were unable to distinguish Hispanic and non-Hispanic respondents for that year. Because Hispanic people tend to have low health care use, failing to exclude them from our White group in 1963 likely biased our estimates toward showing artifactually smaller differences between Black and White (non-Hispanic) respondents that year and would not influence analyses in subsequent years. Finally, although many medical services have shifted from inpatient to ambulatory settings during the past 60 years, our (inflation-adjusted) measures of overall expenditures for services should accurately capture overall time trends.

## Conclusions

The persistence of large racial gaps in the amounts of medical care delivered to White and Black patients in the US suggests that structural racism is ingrained in the health care system. The widening racial-ethnic gap in life expectancy during the COVID-19 pandemic^49^ further highlights the urgency of reforms that promote racial equity. Policies to equalize financial access to care would likely bridge some of the gaps, but additional steps will also be required. Addressing shortages of Black health care professionals and managers, investing in Black-serving medical facilities, increasing community outreach efforts, and enacting other measures that help earn Black patients’ trust in the health care system could also promote equity. Healing the persistent racial divide in medical care in the US would contribute to and benefit from measures to mend the social and economic schisms of race.

## Notes

Supplement. **eTable 1.** Black-White Disparities in Visits, Inpatient Days and Expenditures, 1963-2019 **eTable 2.** Association Between Annual per Capita Health Care Utilization and Race, Adjusted for Demographic and Health Status Differences 2014-2019 **eTable 3.** Association Between Annual per Capita Health Care Utilization and Race, by Gender, 2014-2019 **eTable 4.** Quantile Regression Analysis of White-Black Differences in per Capita Ambulatory Visits and Total Health Expenditures, 2014-2019 **eTable 5.** Alternative Regression Specification for Expenditures: Association Between Natural Logarithm of Expenditures and Race, Adjusted for Age and Sex, 2014-2019
