# Supplementary material for: Trends in Health Care Use Among Black and White Persons in the US, 1963-2019
Source: JAMA Netw Open. 2022 Jun 14;5(6):e2217383. doi: 10.1001/jamanetworkopen.2022.17383 (PMC9198752; doi:10.1001/jamanetworkopen.2022.17383)
Supplement: Supplement. — eTable 1. Black-White Disparities in Visits, Inpatient Days and Expenditures, 1963-2019 eTable 2. Association Between Annual per Capita Health Care Utilization and Race, Adjusted for Demographic and Health Status Differences 2014-2019 eTable 3. Association Between Annual per Capita Health Care Utilization and Race, by Gender, 2014-2019 eTable 4. Quantile Regression Analysis of White-Black Differences in per Capita Ambulatory Visits and Total Health Expenditures, 2014-2019 eTable 5. Alternative Regression Specification for Expenditures: Association Between Natural Logarithm of Expenditures and Race, Adjusted for Age and Sex, 2014-2019 [file jamanetwopen-e2217383-s001.pdf]

Supplementary Online Content

Dickman SL, Gaffney A, McGregor A, et al. Trends in health care use among Black and White persons in the US, 1963-2019. *JAMA Netw Open*. 2022;5(6):e2217383. doi:10.1001/jamanetworkopen.2022.17383

eTable 1. Black-White Disparities in Visits, Inpatient Days and Expenditures, 1963-2019

eTable 2. Association Between Annual per Capita Health Care Utilization and Race, Adjusted for Demographic and Health Status Differences 2014-2019

eTable 3. Association Between Annual per Capita Health Care Utilization and Race, by Gender, 2014-2019

eTable 4. Quantile Regression Analysis of White-Black Differences in per Capita Ambulatory Visits and Total Health Expenditures, 2014-2019

eTable 5. Alternative Regression Specification for Expenditures: Association Between Natural Logarithm of Expenditures and Race, Adjusted for Age and Sex, 2014-2019

This supplementary material has been provided by the authors to give readers additional information about their work.

© 2022 Dickman SL et al. *JAMA Network Open.*

eTable 1: Black-White disparities in visits, inpatient days and expenditures, 1963-2019

			Annual utilization per capita (95% CI)						
			1960s	1970s	1980s	1990s	2000s	2010s	
								2010 - 2013	2014 - 2019
Ambulatory visits (#)									
	Total	White	4.0 (3.8 - 4.1)	3.4 (3.3 - 3.4)	5.3 (5.1 - 5.5)	5.7 (5.5 - 5.8)	6.4 (6.3 - 6.5)	6.7 (6.5 - 6.9)	7.9 (7.7 – 8.1)
		Black	2.8 (2.4 - 3.2)	2.6 (2.5 - 2.7)	3.7 (3.5 - 4.0)	3.3 (3.1 - 3.5)	3.9 (3.7 - 4.1)	4.7 (3.9 - 4.4)	4.7 (4.5 - 5.0)
		W:B ratio	1.43	1.31	1.43	1.73	1.64	1.63	1.68
	Privately insured 18-64	White	5.0 (4.7 - 5.4)	3.6 (3.5 - 3.6)	5.2 (5.0 - 5.5)	5.7 (5.5 - 5.8)	6.4 (6.2 - 6.5)	6.3 (6.1 - 6.6)	6.9 (6.7 - 7.1)
		Black	3.9 (3.1 - 4.6)	3.1 (2.9 - 3.2)	4.1 (3.6 - 4.7)	3.7 (3.3 - 4.1)	4.2 (3.9 - 4.4)	4.1 (3.7 - 4.4)	4.3 (4.1 - 4.6)
		W:B ratio	1.28	1.16	1.27	1.54	1.52	1.54	1.60
	Medicaid 18-64	White	N/A	6.7 (5.9 - 7.5)	9.6 (8.3 - 10.9)	10.6 (9.4 - 11.9)	11.6 (10.7 - 12.4)	10.3 (9.4 - 11.2)	11.6 (10.6 - 12.6)
		Black	-	6.2 (5.0 - 7.3)	7.2 (5.5 - 8.9)	7.1 (5.4 - 8.8)	7.9 (7.0 - 8.7)	8.2 (7.0 - 9.5)	7.7 (6.8 - 8.6)
		W:B ratio	-	1.09	1.33	1.50	1.47	1.25	1.51
Dental visits (#)									
	Total	White	N/A	1.49 (1.46 - 1.51)	1.44 (1.40 - 1.48)	1.25 (1.22 - 1.27)	1.22 (1.20 - 1.24)	1.08 (1.04 - 1.11)	1.16 (1.14 - 1.19)
		Black	-	0.70 (0.66 - 0.73)	0.66 (0.61 - 0.72)	0.56 (0.53 - 0.59)	0.60 (0.57 - 0.62)	0.58 (0.55 - 0.62)	0.62 (0.59 - 0.65)
		W:B ratio	-	2.13	2.18	2.23	2.03	1.86	1.87
	Privately insured 18-64	White	-	1.65 (1.62 - 1.69)	1.40 (1.35 - 1.46)	1.36 (1.33 - 1.39)	1.33 (1.31 - 1.36)	1.14 (1.11 - 1.18)	1.15 (1.12 - 1.18)
		Black	-	0.91 (0.84 - 0.98)	0.71 (0.62 - 0.80)	0.81 (0.74 - 0.87)	0.79 (0.75 - 0.84)	0.76 (0.70 - 0.81)	0.75 (0.71 - 0.80)
		W:B ratio	-	1.81	1.97	1.68	1.68	1.50	1.53
	Medicaid 18-64	White	-	0.98 (0.72 - 1.24)	1.12 (0.87 - 1.36)	0.83 (0.72 - 0.94)	0.84 (0.76 - 0.91)	0.71 (0.59 - 0.84)	0.79 (0.71 - 0.86)
		Black	-	0.78 (0.35 - 1.22)	0.68 (0.47 - 0.89)	0.58 (0.46 - 0.7)	0.55 (0.47 - 0.62)	0.43 (0.36 - 0.5)	0.46 (0.42 - 0.5)
		W:B ratio	-	1.26	1.65	1.43	1.53	1.65	1.72
Emergency visits (#)									
	Total	White	N/A	0.20 (0.19 - 0.21)	0.24 (0.23 - 0.25)	0.17 (0.16 - 0.17)	0.19 (0.18 - 0.19)	0.19 (0.18 - 0.20)	0.21 (0.20 - 0.22)
		Black	-	0.23 (0.19 - 0.27)	0.33 (0.30 - 0.35)	0.18 (0.17 - 0.20)	0.23 (0.22 - 0.24)	0.24 (0.23 - 0.26)	0.25 (0.24 - 0.26)
		W:B ratio	-	0.87	0.73	0.94	0.83	0.79	0.84
	Privately insured 18-64	White	-	0.17 (0.16 - 0.18)	0.20 (0.18 - 0.21)	0.13 (0.12 - 0.14)	0.13 (0.13 - 0.14)	0.13 (0.12 - 0.14)	0.13 (0.12 - 0.14)
		Black	-	0.23 (0.19 - 0.28)	0.31 (0.26 - 0.36)	0.14 (0.12 - 0.16)	0.17 (0.16 - 0.18)	0.17 (0.15 - 0.19)	0.17 (0.15 - 0.18)
		W:B ratio	-	0.74	0.65	0.93	0.76	0.76	0.76

© 2022 Dickman SL et al. *JAMA Network Open.*

	**Medicaid 18-64**	**White**	-	0.46 (0.35 - 0.56)	0.54 (0.44 - 0.64)	0.47 (0.39 - 0.54)	0.52 (0.48 - 0.56)	0.51 (0.45 - 0.57)	0.48 (0.45 - 0.52)
		**Black**	-	0.51 (0.34 - 0.68)	0.59 (0.41 - 0.76)	0.36 (0.30 - 0.42)	0.45 (0.40 - 0.49)	0.46 (0.40 - 0.52)	0.46 (0.42 - 0.49)
		**W:B ratio**	-	0.90	0.92	1.31	1.16	1.11	1.04
**Inpatient days (#)**									
	**Total**	**White**	N/A	1.01 (0.93 - 1.10)	0.90 (0.79 - 0.95)	0.57 (0.54 - 0.61)	0.57 (0.54 - 0.60)	0.53 (0.48 - 0.57)	0.49 (0.46 - 0.53)
		**Black**	-	1.20 (0.98 - 1.42)	1.10 (0.95 - 1.24)	0.59 (0.51 - 0.67)	0.72 (0.67 - 0.77)	0.63 (0.54 - 0.71)	0.58 (0.51 - 0.64)
		**W:B ratio**	-	0.84	0.82	0.97	0.79	0.84	0.84
	**Privately insured 18-64**	**White**	-	0.51 (0.43 - 0.58)	0.51 (0.43 - 0.58)	0.29 (0.25 - 0.34)	0.26 (0.24 - 0.28)	0.26 (0.23 - 0.29)	0.19 (0.17 - 0.21)
		**Black**	-	0.78 (0.50 - 1.06)	0.78 (0.48 - 1.07)	0.32 (0.24 - 0.39)	0.35 (0.30 - 0.39)	0.29 (0.22 - 0.36)	0.26 (0.20 - 0.33)
		**W:B ratio**	-	0.65	0.65	0.91	0.74	0.90	0.73
	**Medicaid 18-64**	**White**	-	2.57 (1.63 - 3.51)	1.80 (1.14 - 2.46)	1.68 (1.25 - 2.11)	1.54 (1.25 - 1.83)	1.25 (0.94 - 1.55)	1.16 (0.92 - 1.40)
		**Black**	-	3.00 (2.13 - 3.86)	2.00 (1.24 - 2.77)	1.48 (1.04 - 1.91)	1.62 (1.3 - 1.95)	1.21 (0.98 - 1.45)	1.01 (0.76 - 1.26)
		**W:B ratio**	-	0.86	0.90	1.14	0.95	1.07	1.15
**Total health spending ($)**									
	**Total**	**White**	739 (698 - 781)	1,470 (1,431 - 1,510)	2,741 (2,608 - 2,873)	3,491 (3,371 - 3,612)	4,826 (4,712 - 4,939)	5,661 (5,470 - 5,852)	6,714 (6,519 - 6,908)
		**Black**	378 (284 - 472)	1,169 (1,074 - 1,264)	2,532 (2,299 - 2,765)	2,391 (2,191 - 2,591)	3,609 (3,445 - 3,773)	4,332 (4,043 - 4,620)	4,834 (4,568 - 5,101)
		**W:B ratio**	1.96	1.26	1.08	1.46	1.34	1.31	1.39
	**Privately insured 18-64**	**White**	1,056 (972 - 1,140)	1,580 (1,532 - 1,627)	2,383 (2,221 - 2,546)	3,072 (2,916 - 3,229)	4,480 (4,325 - 4,634)	5,394 (5,127 - 5,661)	5,902 (5,648 - 6,155)
		**Black**	883 (526 - 1,241)	1,622 (1,449 - 1,794)	2,414 (2,062 - 2,767)	2,186 (1,912 - 2,460)	3,509 (3,292 - 3,725)	4,432 (3,882 - 4,982)	4,611 (4,134 - 5,088)
		**W:B ratio**	1.20	0.97	0.99	1.41	1.28	1.22	1.28
	**Medicaid 18-64**	**White**	N/A	3,918 (2,905 - 4,931)	5,116 (4,025 - 6,208)	7,398 (6,361 - 8,435)	10,299 (8,641 - 11,957)	8,904 (7,854 - 9,955)	9,665 (8,812 - 10,518)
		**Black**	-	4,429 (3,071 - 5,788)	4,630 (2,984 - 6,277)	5,400 (4,266 - 6,534)	7,663 (6,728 - 8,598)	8,288 (7,362 - 9,215)	7,822 (6,801 - 8,843)
		**W:B ratio**	-	0.88	1.10	1.37	1.34	1.07	1.24

Notes:
Data sources: 1963 and 1970 Surveys of Health Services Utilization and Expenditures; 1977 and 1980 National Medical Care Utilization and Expenditure Surveys; 1987 National Medical Expenditure Survey; 1996-2019 Medical Expenditure Panel Surveys.
Differences indicate annual per-capita rates, Black minus White.
Confidence intervals are adjusted for complex survey design using SAS 9.4 *survey* procedures.

© 2022 Dickman SL et al. *JAMA Network Open.*

eTable 2: Association between annual per capita health care utilization and race, by gender, 2014-2019

	Adjusted per capita difference [a]: rate for Black people relative to White people (95% CI)	
	**Men**	**Women**
**Ambulatory visits (#)**	-2.18 (-2.70, -1.66)	-3.12 (-3.46, -2.77)
**Dental visits (#)**	-0.49 (-0.53, -0.44)	-0.55 (-0.61, -0.49)
**Emergency visits (#)**	0.03 (0.02, 0.05)	0.07 (0.05, 0.09)
**Inpatient days (#)**	0.16 (0.00, 0.32)	0.12 (-0.02, 0.25)
**Total health expenditures ($)**	-$900 (-$1,353, -$448)	-$1,158 (-$1,594, -$722)

Notes:
Data are pooled from 2014 – 2019 Medical Expenditures Panel Surveys
Confidence intervals are adjusted for complex survey design using SAS 9.4 *survey* procedures
Negative binomial regressions used for count outcomes (e.g. visits) and linear regression for expenditures
[a] Differences indicate annual per-capita rates, Black minus White, adjusted for age (in years).

© 2022 Dickman SL et al. *JAMA Network Open.*

eTable 3: Association between annual per capita health care utilization and race, adjusted for demographic and health status differences 2014-2019

	Adjusted per capita difference [a]: Black minus White (95% CI)		
	**Total population**	**Adults aged 18-64**	**Privately insured adults aged 18-64**
**Ambulatory visits (#)**	-3.06 (-3.37, -2.75)	-2.72 (-3.05, -2.38)	-2.92 (-3.29, -2.55)
**Dental visits (#)**	-0.51 (-0.55, -0.47)	-0.42 (-0.46, -0.37)	-0.38 (-0.44, -0.32)
**Emergency visits (#)**	0.04 (0.03, 0.05)	0.06 (0.05, 0.08)	0.03 (0.01, 0.05)
**Inpatient days (#)**	0.09 (0.00, 0.18)	0.10 (0.03, 0.17)	0.06 (-0.01, 0.13)
**Total health expenditures ($)**	-$1,457 (-$1,762, -$1,151)	-$1,327 (-$1,715, -$939)	-$1,514 (-$2,018, -$1,010)

Notes:
Data are pooled from 2014 – 2019 Medical Expenditures Panel Surveys
Confidence intervals are adjusted for complex survey design using SAS 9.4 *survey* procedures
Negative binomial regressions used for count outcomes (e.g. visits) and linear regression for expenditures
[a] Differences are adjusted for age (in years), sex (male, female), and self-reported health status (poor, fair, good, very good, excellent) using SAS 9.4 *survey* procedures

© 2022 Dickman SL et al. *JAMA Network Open.*

eTable 4: Quantile regression analysis of White-Black differences in per capita ambulatory visits and total health expenditures, 2014-2019

	Adjusted difference [a]: Black individuals minus White individuals (95% CI)				
	**50th percentile**	**75th percentile**	**90th percentile**	**95th percentile**	**97.5th percentile**
**Ambulatory visits (#)**	-1.42 (-1.48, -1.38)	-3.00 (-3.11, -2.89)	-5.69 (-5.95, -5.42)	-8.33 (-8.79, -7.87)	-11.34 (-12.50, -10.18)
**Total health spending ($)**	-634 (-661, -607)	-1,255 (-1,322, -1,188)	-2,601 (-2,803, -2,398)	-3,570 (-3,973, -3,166)	-4,779 (-5,783, -3,775)

Notes:
Data sources: 2014-2019 Medical Expenditure Panel Surveys.
All results are significant at $p < 0.05$
Figures represent adjusted differences in annual visit numbers and total expenditures, Black minus White, at each quantile of healthcare utilization
[a] Differences are adjusted for age (in years), sex (male, female), and self-reported health status (poor, fair, good, very good, excellent) using SAS 9.4 *survey* procedures.

© 2022 Dickman SL et al. *JAMA Network Open.*

eTable 5: Alternative regression specification for expenditures: association between natural logarithm of expenditures and race, adjusted for age and sex, 2014-2019

	Total population	Adults aged 18-64	Privately insured adults aged 18-64	Medicaid adults aged 18-64
**Race (Black-White) coefficient (SE)**	-1.01[a] (0.04)	-1.03 [a] (0.05)	-0.99 [a] (0.06)	-0.72 [a] (0.11)

Notes:
Data are pooled from 2014 – 2019 Medical Expenditures Panel Surveys
Standard errors (in parentheses) are adjusted for complex survey design using SAS 9.4 *survey* procedures
[a] indicates difference is significant at $p<0.01$

© 2022 Dickman SL et al. *JAMA Network Open.*
